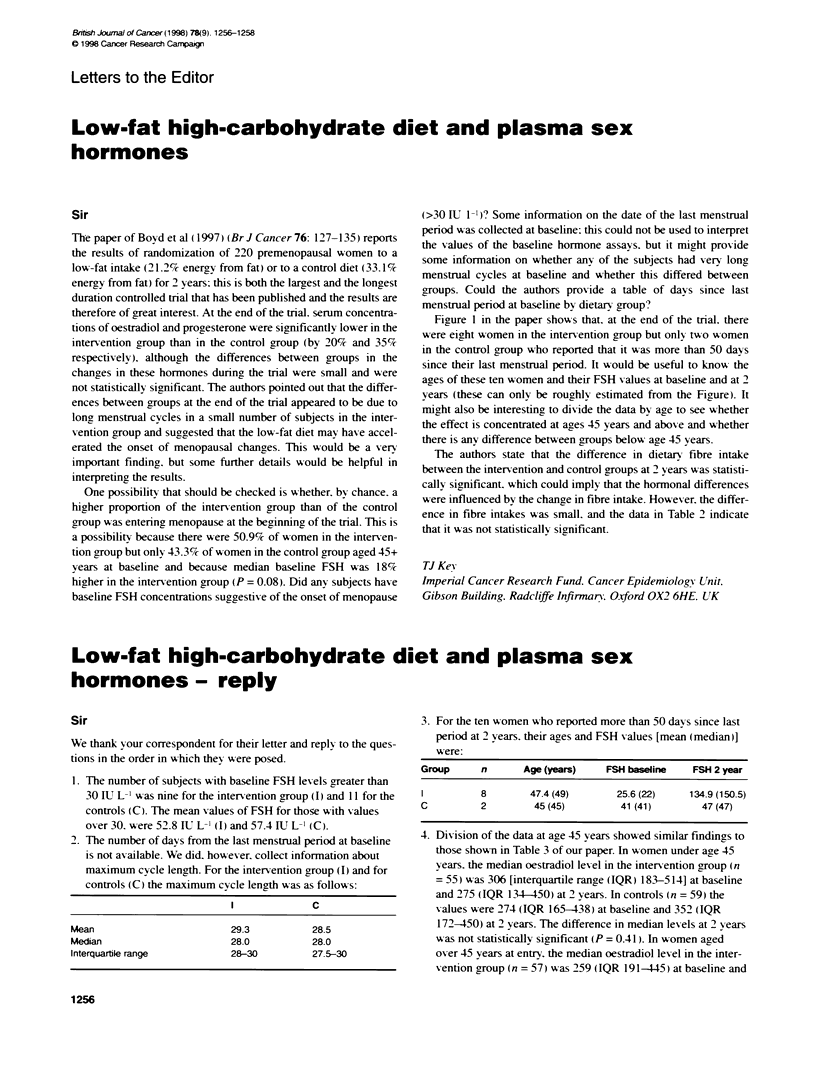# Low-fat high-carbohydrate diet and plasma sex hormones.

**DOI:** 10.1038/bjc.1998.664

**Published:** 1998-11

**Authors:** T. J. Key


British Journal of Cancer (1998) 78(9). 1256–1258

© 1998 Cancer Research Campaign

Letters to the Editor

### Low-fat high-carbohydrate diet and plasma sex hormones

### Sir

The paper of Boyd et al (1997) (*Br J Cancer* **76**: 127–135) reports the results of randomization of 220 premenopausal women to a low-fat intake (21.2% energy from fat) or to a control diet (33.1% energy from fat) for 2 years: this is both the largest and the longest duration controlled trial that has been published and the results are therefore of great interest. At the end of the trial. serum concentrations of oestradiol and progesterone were significantly lower in the intervention group than in the control group (by 20% and 35% respectively). although the differences between groups in the changes in these hormones during the trial were small and were not statistically significant. The authors pointed out that the differences between groups at the end of the trial appeared to be due to long menstrual cycles in a small number of subjects in the intervention group and suggested that the low-fat diet may have accelerated the onset of menopausal changes. This would be a very important finding. but some further details would be helpful in interpreting the results.

One possibility that should be checked is whether. by chance. a higher proportion of the intervention group than of the control group was entering menopause at the beginning of the trial. This is a possibility because there were 50.9% of women in the intervention group but only 43.3% of women in the control group aged 45+ years at baseline and because median baseline FSH was 18% higher in the intervention group ($P = 0.08$). Did any subjects have baseline FSH concentrations suggestive of the onset of menopause (>30 IU $l^{-1}$)? Some information on the date of the last menstrual period was collected at baseline: this could not be used to interpret the values of the baseline hormone assays. but it might provide some information on whether any of the subjects had very long menstrual cycles at baseline and whether this differed between groups. Could the authors provide a table of days since last menstrual period at baseline by dietary group?

Figure 1 in the paper shows that. at the end of the trial. there were eight women in the intervention group but only two women in the control group who reported that it was more than 50 days since their last menstrual period. It would be useful to know the ages of these ten women and their FSH values at baseline and at 2 years (these can only be roughly estimated from the Figure). It might also be interesting to divide the data by age to see whether the effect is concentrated at ages 45 years and above and whether there is any difference between groups below age 45 years.

The authors state that the difference in dietary fibre intake between the intervention and control groups at 2 years was statistically significant. which could imply that the hormonal differences were influenced by the change in fibre intake. However. the difference in fibre intakes was small. and the data in Table 2 indicate that it was not statistically significant.

*TJ Key*
*Imperial Cancer Research Fund. Cancer Epidemiology Unit. Gibson Building. Radcliffe Infirmary. Oxford OX2 6HE. UK*